# Interleukin-6 versus Common Inflammatory Biomarkers for Diagnosing Fracture-Related Infection: Utility and Potential Influencing Factors

**DOI:** 10.1155/2021/1461638

**Published:** 2021-09-20

**Authors:** Xing-qi Zhao, Hao-yang Wan, Han-jun Qin, Nan Jiang, Bin Yu

**Affiliations:** ^1^Division of Orthopaedics and Traumatology, Department of Orthopaedics, Nanfang Hospital, Southern Medical University, Guangzhou 510515, China; ^2^Guangdong Provincial Key Laboratory of Bone and Cartilage Regenerative Medicine, Nanfang Hospital, Southern Medical University, Guangzhou 510515, China

## Abstract

Currently, the utility of white blood cell count (WBC), erythrocyte sedimentation rate (ESR), and C-reactive protein (CRP), for diagnosis of fracture-related infection (FRI), is still controversial, and potential efficiency of interleukin-6 (IL-6) as a novel cytokine in assisted diagnosis of FRI remains unclear. This study is aimed at investigating the utility and potential influencing factors of IL-6 and the common biomarkers for diagnosing FRI. Preoperative serological levels of IL-6 and the three biomarkers were compared between 407 FRI patients and 195 fracture-healed (FH) patients. Diagnostic efficiency of the indicators was evaluated using the areas under the receiver operating characteristic (ROC) curves, and their potential influencing factors were also analyzed. Outcomes showed that the median levels of all of the four biomarkers were significantly higher among the FRI patients than those among the FH patients (*P* < 0.01). The areas below the ROC curves of ESR, CRP, and IL-6 were 76.5%, 76.4%, and 71.8%, respectively, with WBC of only 56.9%. Compared with ESR and CRP, IL-6 displayed a lower sensitivity (ESR vs. CRP vs. IL − 6 = 72.7% vs. 65.6% vs. 57.5%) but a higher specificity (ESR vs. CRP vs. IL − 6 = 70.3% vs. 75.4% vs. 83.6%). Serological IL-6 level was influenced by pathogen culture result and pathogen number; nonetheless, bacteria type appeared to have no influence on the levels of the four biomarkers. In short, this study displayed similar value of IL-6 with that of ESR and CRP in assisted diagnosis of FRI. Whether IL-6 can be regarded as a promising diagnostic indicator requires more studies.

## 1. Introduction

Fracture-related infection (FRI) refers to the osseous infection with or without surrounding soft tissue infection following trauma and orthopaedic surgery [1]. The average cost of a single FRI treatment exceeds US $15,000, four times as that of periprosthetic joint infection (PJI) [2]. Currently, successful FRI management poses great challenges on orthopaedic surgeons as the risk of infection recurrence could achieve as high as 20% to 30% [3], and therefore, brings great pressures, not only to the patients [4] but also to their families and medical insurance and care systems [5]. Early and accurate diagnosis is essential, paving the way for subsequent treatment. However, when there are no typical signs prior to surgery, such as sinus tract or purulent discharge, FRI diagnosis can only be made during or even after surgery, such as intraoperative findings of infection clues, bacterial culture, and histopathological test [6]. Thus, there exists a risk of misdiagnosis, resulting in unsatisfying efficacy.

As an important aspect of early diagnosis, detection of serological levels of inflammatory biomarkers displays advantages, which is noninvasive, cheap, and capable of reflecting the host immune status quickly. A previous study had indicated that when the levels of the common inflammatory biomarkers, including white blood cell count (WBC), erythrocyte sedimentation rate (ESR), and C-reactive protein (CRP), exceeded their normal upper limits, the predicted probability of infected nonunion could increase up to 100% [7]. In addition, such common biomarkers have been routinely applied in assisted diagnosis of PJI [8]. However, recently, several investigations pointed out their limited values in diagnosis of FRI owing to their not-so-high sensitivities as well as specificities [6, 9–11]. All the abovementioned issues suggested that there still exist disputes regarding the utility of such inflammatory indicators for FRI diagnosis, and therefore, it is quite necessary that more investigations should be performed to solve the controversies, and moreover, potential novel inflammation cytokines should also be explored.

Interleukin-6 (IL-6) acts as a pleiotropic cytokine with multiple roles in immune response, tissue repair, and regeneration. Rapid generation of IL-6 contributes to the host defense during tissue injury and infection, while excessive synthesis and massive accumulation of IL-6 often lead to disease pathology [12]. Recent studies found IL-6 elevation in COVID-19 patients and IL-6 antagonists might bring benefits to the patients with severe COVID-19 symptoms [13, 14]. However, up till now, the number of clinical investigations focused on the role of IL-6 in FRI diagnosis remains limited. Our previous preliminary report indicated that the positive rate of IL-6 achieved 60.36% in patients with extremity chronic osteomyelitis (COM) [15]. However, due to the retrospective and observational design, as well as the lack of controls, the level of evidence was limited. Additionally, many factors that may influence levels of the biomarkers were also not analyzed. Therefore, in order to better evaluate potential role of IL-6 in assisted diagnosis of FRI, well-designed studies are the premise.

In view of the existed disputes regarding the common inflammatory biomarkers in FRI diagnosis and indefinite role of IL-6 as well, we performed the present study, with the aim of summarizing the utility and potential influencing factors of IL-6 together with the traditional biomarkers for assisted diagnosis of FRI.

## 2. Patients and Methods

### 2.1. Study Design, Setting, and Participants

This study, designed as a retrospective controlled analysis, was conducted in Southern Medical University Nanfang Hospital, a tertiary healthcare center providing specialist treatment to patients with musculoskeletal infections. Study population of the both groups was selected from the electronic medical record (EMR) system. Participants of the FRI group were patients who had sought medical attention for FRI between January 1st, 2010, and September 1st, 2019, with eligible data for analysis. The controlled group, as fracture healed (FH) group, was constituted of patients who had required removal of the orthopaedic implants after being confirmed with fracture healed between January 1st, 2018, and September 1st, 2019. The study was performed in accordance with the Declaration of Helsinki and also was approved by the medical ethical committee of the hospital.

### 2.2. Diagnostic Criteria and Inclusion and Exclusion Criteria

FRI diagnosis was made based on the international experts' consensus on FRI diagnosis [1]. Detailed diagnostic criteria and inclusion and exclusion criteria for FRI and FH group participants are listed in Table 1.

### 2.3. Statistical Analysis

The Statistical Product and Service Solutions software (version 20.0, SPSS Inc., Chicago, IL, USA) and R software (version 3.6.3) were used for data analysis. For continuous data, the Shapiro-Wilk normality test was used to test whether the data were distributed normally. For normally distributed data, the sample description was expressed in the form of mean (M) ± standard deviation (SD); the mean differences between or among two or over two groups were compared using the independent sample *t*-test or the one-way analysis of variance (ANOVA), respectively. For the data that did not conform to normal distribution, the data were presented with median and interquartile range (IQR); comparisons between or among two or over two groups were performed using the Wilcoxon rank sum test or the Kruskal-Wallis test. Binary variables were described as percentage, and the comparison of rates among groups was conducted using the chi square test.

To evaluate diagnostic efficiency of the inflammation indicators in FRI, the receiver operating characteristic (ROC) curve was drawn firstly, and then, the diagnostic efficiency of the indicators was assessed according to the area under the curve (AUC). The best cut-off point was obtained by calculating the maximum Youden index (sensitivity + specificity–1), and then, the sensitivity and specificity were calculated according to the critical point. All the statistical test results were two-sided, with *P* value of < 0.05 as statistically significant.

## 3. Results

### 3.1. Demographics and Clinical Characteristics of the Patients Included

Altogether 407 FRI patients and 195 FH patients met their corresponding inclusion criteria and were included for analysis. Demographics and clinical characteristics of the FRI patients and the FH patients are listed in Table 2.

### 3.2. Normal Distribution Evaluation

Shapiro-Wilk normality test was applied to evaluate whether the data regarding levels of the four biomarkers distributed normally. Outcomes revealed that none of such data distributed normally (Table 3).

### 3.3. Comparisons of Serological Levels of Inflammatory Biomarkers between FRI Group and FH Group

**Figure 1:**
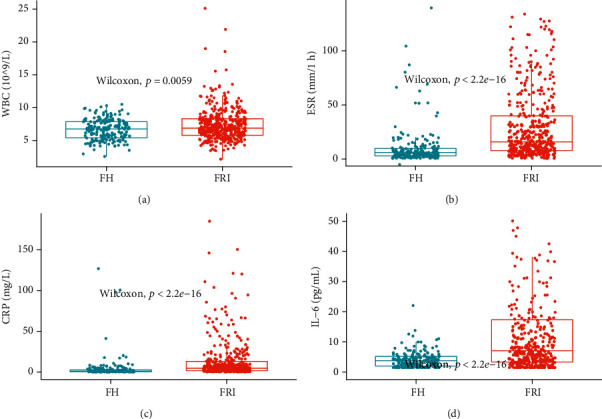
Plots of inflammatory biomarkers' levels in FRI and FH groups: (a) WBC; (b) ESR; (c) CRP; (d) IL-6.

As shown in Figure 1, the median levels of all the four biomarkers were significantly higher in the FRI group than those in the FH group (*P* < 0.01).

### 3.4. Effectiveness of Discrimination of the Inflammatory Biomarkers

**Figure 2:**
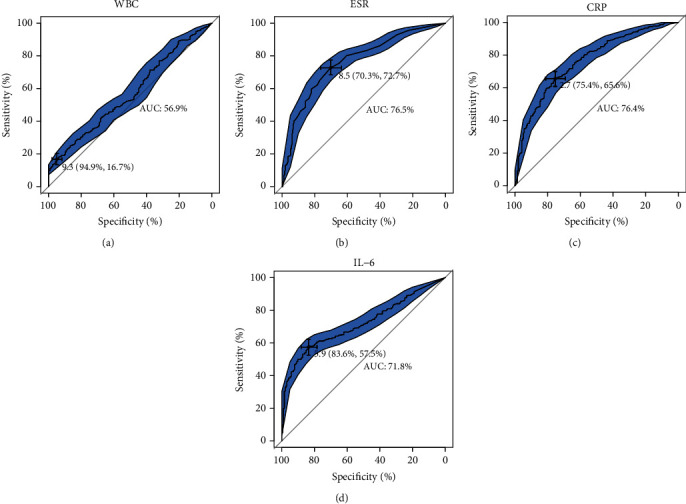
ROCs of the four inflammatory biomarkers for FRI diagnosis. The areas in deep blue represent 95% CIs of the AUCs.

As shown in Figure 2 and Table 4, the AUCs of WBC, ESR, CRP, and IL-6 were 56.9%, 76.5%, 76.4%, and 71.8%, respectively, demonstrating similar diagnostic value of IL-6 with that of ESR and CRP. Furthermore, IL-6 displayed a lower sensitivity but a higher specificity than ESR and CRP at their corresponding optimal cut-off values (Table 4).

### 3.5. Potential Factors That Influencing the Inflammatory Biomarkers' Levels in FRI Patients

**Figure 3:**
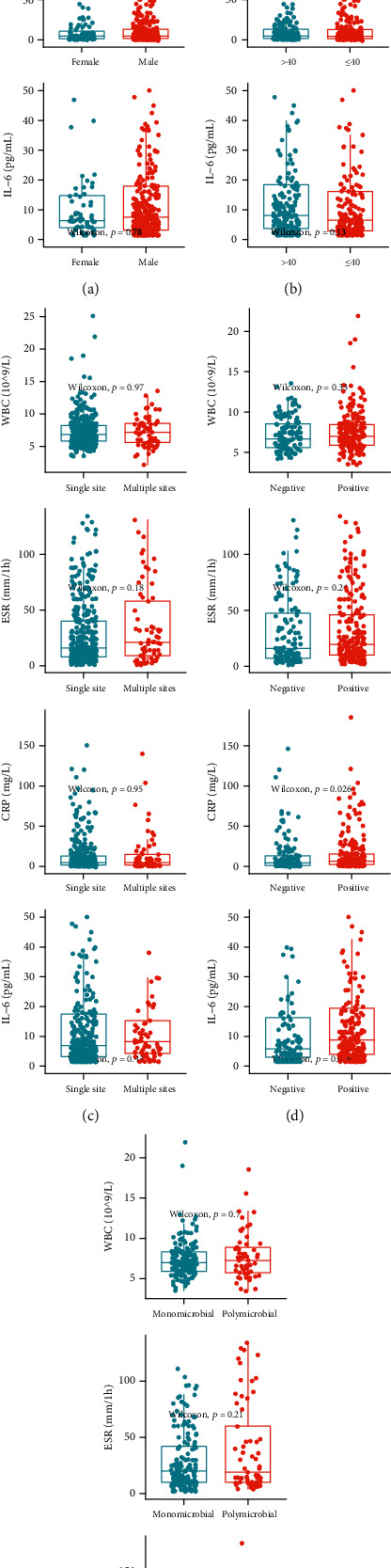
Plots of the inflammatory biomarkers in FRI patients categorized by possible influencing factors. (a) Gender: female vs. male. (b) Onset of age: > 40 vs. ≤ 40 years. (c) Infection site number: single vs. multiple. (d) Tissue culture outcome: negative vs. positive. (e) Pathogen number: monomicrobial vs. polymicrobial.

**Figure 4:**
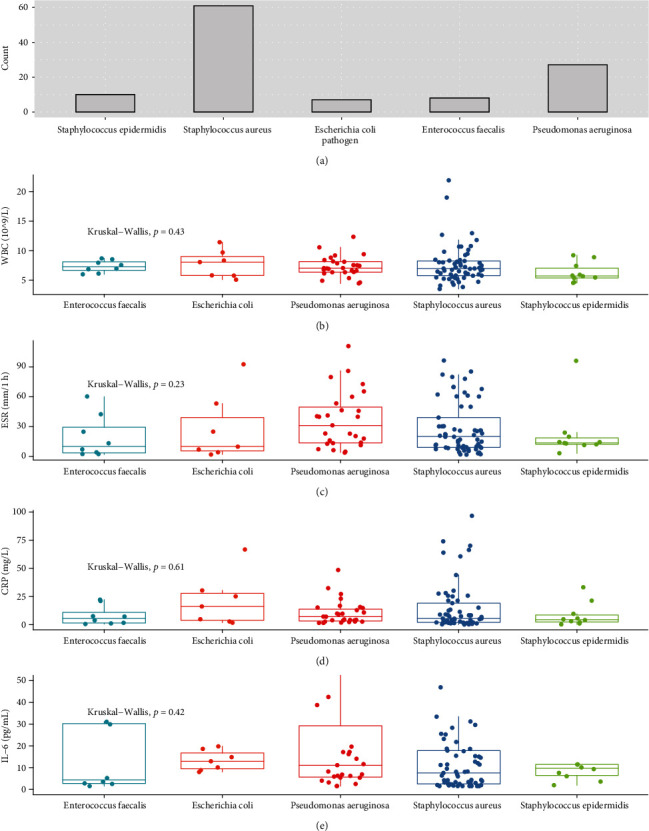
Plots of inflammatory biomarkers' levels among different types of pathogenic bacteria: (a) the top five pathogens accounting for monomicrobial infection; (b) WBC; (c) ESR; (d) CRP; (e) IL-6.

FRI patients were categorized by sex, age, infection site number, culture outcome, and pathogen number. As depicted in Figure 3, WBC value was not influenced by any of the above five factors, while ESR value was only influenced by gender, with female patients having a significantly higher level than the male patients (*P* < 0.001). CRP level was affected by culture outcome, indicating that patients with positive outcomes having a significantly higher level than those with negative ones (*P* = 0.026). IL-6 level was affected by both culture outcome and pathogen number, indicating that FRI patients with positive culture outcomes and polymicrobial infections had significantly higher levels than those with negative outcomes (*P* = 0.016) and monomicrobial infections (*P* = 0.049, Figure 4), separately.

### 3.6. Influence of Bacteria Type on Serological Levels of the Inflammatory Biomarkers

In order to clarify whether levels of the inflammatory indicators were affected by pathogen type, comparisons were conducted regarding the biomarkers' levels among the top frequently detected bacteria, and outcomes showed that bacterial type did not affect serological levels of the four inflammatory indicators (Figure 4).

## 4. Discussion

Currently, as one of the most catastrophic complications after trauma and orthopaedic surgery, FRI represents great challenges in front of orthopaedic surgeons. On one hand, early and accurate diagnosis is sometimes difficult as clinical manifestations of some patients are untypical. On the other hand, successful treatment is usually intractable, which not only requires complete eradication of infection but also restores limb function, enabling patients being back to work and daily life. Among these issues, early and accurate diagnosis is of great importance, which is the premise of therapy. According to the recently achieved international consensus on FRI [17], clinical signs and findings before and during surgery, pathogen culture, and histology test can be regarded as confirmatory criteria of FRI. However, in some patients with untypical manifestations, FRI diagnosis can only be made during or even after surgery, which increases the misdiagnosis risk. As an important diagnostic tool of FRI before surgery, serological levels of inflammatory biomarkers act as an irreplaceable role. While most of the previous studies focused on PJI, currently, the number of reports investigating potential roles of inflammatory biomarkers for FRI diagnosis remains limited. Thus, we conducted this study to summarize the utility of IL-6 with WBC, ESR, and CRP for FRI diagnosis. In addition, potential factors that may influence the levels of such indicators were also explored.

Based on the retrospective analysis of 602 participants, we found that in this Chinese cohort, IL-6 shared similar diagnostic efficiency with that of ESR and CRP, better than that of WBC. Regarding the influencing factors, levels of both IL-6 and CRP might be influenced by culture outcome; however, aside from culture outcome, pathogen number might also affect IL-6 level. In short, this study suggested that IL-6 possessed similar value with ESR and CRP in assisted diagnosis of FRI.

Although the common inflammatory biomarkers have been widely used for FRI diagnosis, however, disputes still exist regarding the diagnostic efficiency. In 2013, a retrospective controlled study indicated that both ESR and CRP were independently accurate predictors of bone infection, and the predicted probability in case of elevations in WBC, ESR, and CRP could achieve 100% [7]. Later in 2017, another study also obtained similar conclusions [18]. While in a recent cohort study, Brinker and the colleagues indicated that WBC, ESR, and CRP were not significant predicators of osseous infection [11]. The contrasting conclusions arising from these studies may attribute to several reasons, such as different inclusion and diagnostic criteria, and different sample size.

The controversial outcomes and conclusions never mean useless of the routine inflammatory indicators in FRI diagnosis. Outcomes of a recent systematic review and meta-analysis [9] revealed that ESR and CRP lied in the top of specificity and sensitivity for diagnosing FRI, though limited eligible studies were included. The authors concluded that, although sufficient accuracy might be difficult to reach, such biomarkers may be used as a suggestive sign of FRI. That is to say, preoperative levels of such biomarkers may be used as a screen tool to evaluate patient status. Some authors [18] suggested that laboratory analysis of serum inflammatory markers should combine with another tests for FRI diagnosis. Anyhow, inflammatory biomarkers act as an unneglectable role in diagnosis of FRI.

As a novel inflammatory cytokine, IL-6 has been widely used in PJI diagnosis [19–21]; however, its utility in FRI diagnosis is still unclear. Our preliminary report indicated that the positive ratios of ESR and IL-6 lied in the top among the COM patients, suggesting probably definite role of IL-6 in chronic bone infection [15]. While in this cohort, we noticed that IL-6 possessed similar efficiency with ESR and CRP, without more advantages, which is thought-provoking. Undoubtedly, similar to the traditional biomarkers, serum IL-6 level is influenced by both extrinsic and intrinsic factors. Take the disorder itself as an example, FRI is a “high-heterogeneity” disorder, and preoperative levels of inflammatory indicators are influenced by multiple factors, such as infection site, infection duration, pathogen type, and virulence, and even previous treatment. Therefore, in order to more comprehensively evaluate potential role of such cytokines in FRI diagnosis, it is quite necessary to explore their potential influencing factors.

It is interesting that WBC value was not affected by any of the analyzed factors. With respect to ESR, the basal level of ESR of females is higher than that of males, which was also found in the FRI patients. Thus, gender factor should be taken into consideration when utilizing ESR for assisted FRI diagnosis. Regarding CRP and IL-6, they shared similarity that serum levels of them were both influenced by pathogen culture outcome; that is to say, patients with positive culture outcomes had a relatively higher CRP and IL-6 levels. Previous studies had reported that the positive rate of bacterial culture was approximate 70% [22–25]. It is known that negative bacterial culture does not mean no bacterial infection as the culture result is affected by multiple factors, such as bacteria type, culture medium, and culture time. In addition to the culture result, serum IL-6 level was also influenced by pathogen number, with polymicrobial infections displaying a higher IL-6 level than monomicrobial infections. This implies that mixed bacterial infection may produce higher levels of virulence factors and thus a higher level of IL-6.

As mentioned above, different types of pathogens may produce different levels of virulence factors, whether different bacteria lead to different levels of inflammatory biomarkers remains largely unclear. A previous study found that levels of the routine inflammation biomarkers were influenced by the infecting organisms among PJI patients [26]. Similarly, Sigmund et al. also noticed that CRP level caused by a high-virulence pathogen was significantly higher than that of a low-virulence pathogen [6]. While in our study, no significant difference was identified regarding the levels of the four indicators among different bacteria types; however, considering the limited sample size, future studies are warranted to obtain more accurate outcomes.

The current study also has several limitations. First, although the sample size of the present study was larger than most of the previous reports, the imbalance regarding the number of patients between FRI group and FH group may arise bias. Second, the selection of FH as controls may also bring potential risk of bias. Therefore, cautious attitude should be taken towards the outcomes. Third, due to the retrospective design, we only included data available for analysis. It is known that, aside from the reported ones, many other factors may also influence levels of the biomarkers. Therefore, well-designed studies are quite necessary.

## 5. Conclusions

In summary, the present study demonstrated that IL-6 did not present more advantages for FRI diagnosis, which possessed similar efficacy with ESR and CRP. Serological IL-6 level might be influenced by pathogen culture result and pathogen number. Nonetheless, the current study did not find any positive effect of pathogen type on serological levels of the inflammation biomarkers.

## Figures and Tables

**Table 1:** The diagnostic criteria, inclusion and exclusion criteria for FRI group and FH group.

FRI group	FH group
Diagnostic criteria	
According to the consensus of international experts on the diagnosis of FRI, if any of the following four primary diagnostic criteria [1] is met: (1) Formation of sinus or fistula or opening of wound causes bone or implant exposure (2) Purulent exudate in the wound or pus accumulation is found during surgery (3) Positive culture of pathogenic microorganism from deep tissue (4) Positive histopathological test	According to the clinical standard of FH [16], all the following items should be met: (1) No tenderness or longitudinal percussion pain in local part of the fractured site (2) No abnormal local activity (3) X-ray shows continuous callus at the fractured site, and the fracture line has been blurred
Inclusion criteria	
(1) Diagnosed as FRI (2) Patients had available data regarding WBC, ESR, CRP, and IL-6 levels before surgery, which were obtained 2 weeks of antibiotic withdrawal (3) At least one data of the following items should be included: sex, age of onset, injury feature and type, body side and bone site of infection, and culture results of intraoperative samples (4) If the patients had been admitted to the hospital for several times, only the data most related to the first hospitalization of FRI were used	(1) The FH exactly, which met the diagnostic criteria of FH (2) The patient required removal of the fracture fixation devices (3) Patients had data regarding WBC, ESR, CRP, and IL-6 levels before surgery, which were obtained 2 weeks of antibiotic withdrawal (4) The patient was admitted to the hospital only because of FH, without comorbidities
Exclusion criteria	
(1) Bone infection following hematogenous spread or diabetic foot (2) Acute or subacute osteomyelitis (infection symptom < 10 weeks) (3) PJI (4) Unavailable data or missing data	(1) Comorbidities that may influence levels of the biomarkers (2) Unavailable data or missing data

**Table 2:** Demographics and clinical characteristics of 407 FRI patients and 195 FH patients.

Clinical characteristics	FRI group	FH group
Age of first onset (years) median (IQR)^∗^	42 (28, 53)	35 (27, 47)
Male	41 (27, 52)	34.5 (27, 44.75)
Female	46 (34.5, 55.5)	41 (27, 51)
Gender ratio (male/female)	336/71	130/65
Features of injury (no., %)		
Open	251 (61.67%)	18 (9.23%)
Closed	102 (25.06%)	155 (79.49%)
Unavailable	54 (13.27%)	22 (11.28%)
Top 5 injury types (no., %)		
Traffic injury	118 (28.99%)	
Falling injury	52 (12.78%)	
Falling from a height	34 (8.35%)	
Stabbing injury	28 (6.88%)	
Bruise	26 (6.39%)	
Infection side distribution (left/right/bilateral)	209/191/7	
Infection site number (single/multiple)	342/65	
Top 3 infection sites (no., %)		
Tibia	188 (54.97%)	
Femur	65 (19.01%)	
Calcaneus	43 (12.57%)	
Positive rate of pathogen culture	62.31% (210/337)	
Pathogen for infection monomicrobial/polymicrobial	153/57	
Top 5 detected pathogens (no., %)		
*Staphylococcus aureus*	61 (39.87%)	
*Pseudomonas aeruginosa*	27 (17.65%)	
*Staphylococcus epidermidis*	10 (6.53%)	
*Enterococcus faecalis*	8 (5.23%)	
*Escherichia coli*	7 (4.58%)	

^∗^Shapiro-Wilk normality test showed that the data of age was not normally distributed (*W* = 0.99062, *P* < 0.01).

**Table 3:** Distribution tests regarding serological levels of the inflammatory biomarkers.

Inflammatory biomarkers	Shapiro-Wilk *W*	*P* value
WBC (×10^9^/L)	0.87	<0.001
ESR (mm/1 h)	0.74	<0.001
CRP (mg/L)	0.50	<0.001
IL-6 (pg/mL)	0.18	<0.001

**Table 4:** Discriminatory strengths of the four inflammatory biomarkers.

Infection markers	AUC	95% CI	Optimal cut-off value^∗^	Sensitivity	Specificity
WBC (×10^9^/L)	0.569	0.522-0.617	9.3	16.7%	94.9%
ESR (mm/1 h)	0.765	0.725-0.806	8.5	72.7%	70.3%
CRP (mg/L)	0.764	0.724-0.804	2.7	65.6%	75.4%
IL-6 (pg/mL)	0.718	0.678-0.758	5.9	57.5%	83.6%

^∗^The optimal cut-off value was obtained by calculating the maximum Youden index (sensitivity + specificity–1).

## Data Availability

The datasets generated and/or analyzed during this study are not publicly available due to the respect and protection of privacy of the patients but are available from the corresponding authors upon reasonable request.
